# Choroidal Vascular Changes in Acute Idiopathic Maculopathy as Demonstrated by Multimodal Imaging including Optical Coherence Tomography Angiography

**DOI:** 10.1155/2021/6680020

**Published:** 2021-04-06

**Authors:** Tahsin Khundkar, Syed R. Hasan, Mark P. Breazzano, Constance Mei, Brandon B. Johnson

**Affiliations:** ^1^Department of Ophthalmology, University of Minnesota, 516 Delaware Street SE, Minneapolis, MN 55455, USA; ^2^AdvantageCare Physicians, 180-05 Hillside Avenue, Queens, NY 11432, USA; ^3^Department of Ophthalmology, Johns Hopkins University School of Medicine, 87 Thomas Johnson Drive, Frederick, MD 21702, USA; ^4^Department of Ophthalmology, New York Medical College-Jamaica Hospital Medical Center, 134-20 Jamaica Avenue, Richmond Hills, NY 11418, USA; ^5^New York Retina Center, 161 E 32nd St, New York, NY 10016, USA

## Abstract

*Purpose*. To present a case of acute idiopathic maculopathy (AIM) and illustrate primary choroidal perfusion defect using multimodal imaging. *Case Description*. We report a case of a 24-year-old man with a paracentral scotoma of the right eye and recent flu-like illness. The patient was found to have a unilateral ovoid-shaped, placoid lesion just inferior to the fovea. Multimodal imaging confirmed findings most consistent with a diagnosis of acute idiopathic maculopathy (AIM). Serologic studies confirmed a strongly positive immunoglobulin G (IgG) titer for coxsackievirus A. Spectral-domain optical coherence tomography angiography (SD-OCTA) showed bilateral areas of vascular reduction at the level of the choriocapillaris and choroid, sparing the retinal circulation. *Conclusions and Importance*. The changes in outer retina and retinal pigment epithelium, classically described in AIM, are likely secondary to choroidal hypoperfusion.

## 1. Introduction

Acute idiopathic maculopathy (AIM) is a rare, inflammatory disorder classically thought to involve the retinal pigment epithelium (RPE) and outer retina [1]. AIM was initially described by Yannuzzi et al. [1] in a group of healthy, young adults who experienced sudden, unilateral loss of central vision following a flu-like illness with neurosensory retinal detachment and focal, yellow-white thickening at the level of the RPE. The clinical spectrum of the condition was later expanded to include bilateral disease [2]. Coxsackievirus seropositivity on laboratory testing has been described in association with AIM [2, 3]. Most patients experience spontaneous recovery over several weeks to months; however, the use of systemic steroids may hasten recovery [1, 4]. We describe a case of AIM with bilateral, asymmetric findings using multimodal imaging.

## 2. Case Presentation

A 24-year-old, healthy, Caucasian man presented with a “blurred spot” in the vision of his right eye for two days. There were no other ocular complaints. The patient endorsed a recent flu-like episode 12 weeks prior to presentation.

**Figure 1:**
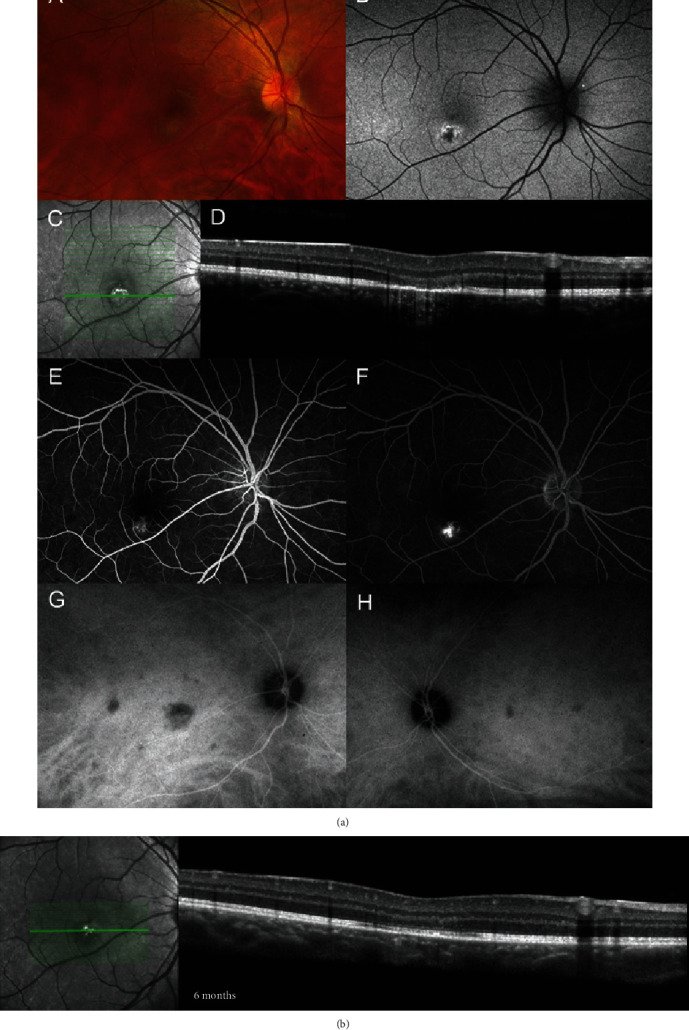
Wide-field fundus photography of the right eye (A) shows an ovoid, hypopigmented placoid lesion adjacent to the fovea. FAF (B) reveals corresponding hypoautofluorescence and surrounding areas of hyperautofluorescence with irregular borders. Near-infrared reflectance (C) and spectral-domain optical coherence tomography (D) reveal speckled hyperreflectivity within the lesion and significant disruption of the outer retina, including the outer nuclear layer, ellipsoid zone, and retinal pigmented epithelium with choroidal hypertransmission. Early hyperfluorescence with FA (E) precedes increased hyperfluorescence of the macular lesion with discrete borders upon recirculation phase (F). Late-phase indocyanine green angiography shows multiple areas of hypocianescence in the right (G) greater than the left eye (H), including the area corresponding to the lesion. (b) Spectral-domain optical coherence tomography at 6 months after initial presentation shows improved attenuation in the outer plexiform layer.

On examination, best-corrected visual acuity was 20/20 in each eye. No relative afferent pupillary defect was observed. Amsler grid testing revealed a superior wedge-shaped paracentral scotoma in the right eye. The anterior segment examination and intraocular pressure were normal in both eyes. No cells in the vitreous were observed. Fundus examination revealed an ovoid-shaped, tan, hypopigmented lesion just inferior to the fovea in the right eye (Figure 1).

Fundus autofluorescence (FAF) showed a corresponding wedge-shaped area of hypoautofluorescence surrounded by a margin of hyperautofluorescence. Near-infrared reflectance (NIR) showed a stippled hyperreflective lesion with a hyporeflective border. Corresponding cross-sectional imaging with spectral-domain optical coherence tomography (SD-OCT) revealed choroidal hypertransmission and outer retinal attenuation from the outer plexiform layer to the retinal pigment epithelium. Fluorescein angiography (FA) of the right eye demonstrated a hyperfluorescent macular lesion and increasing hyperfluorescence mostly within the margins upon recirculation. Late-phase indocyanine green angiography (ICG) showed multiple areas of hypocianescence corresponding to choroidal nonperfusion—worse on the right, but present in both eyes (Figure 1).

**Figure 2:**
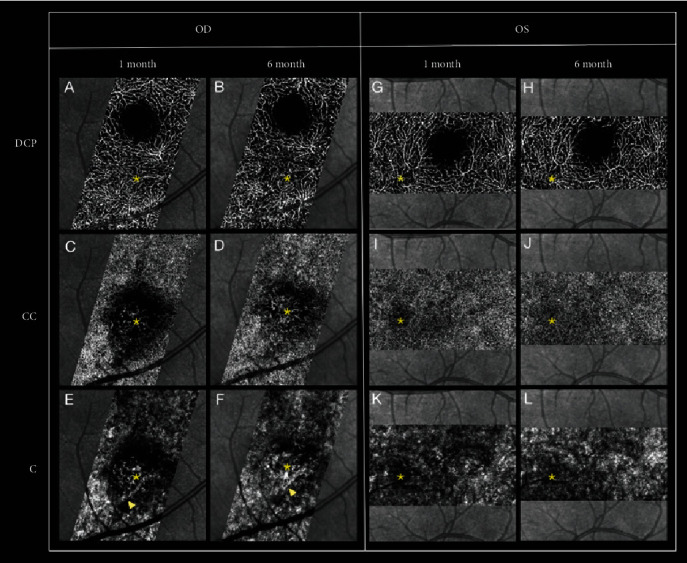
Spectral domain-optical coherence tomography angiography findings at various levels during the 1-month and 6-month follow-up. An asterisk marks the center of the lesion. Normal perfusion is seen in the deep capillary plexus (DCP) of the right eye at 1 month (A) and at 6-month (B) follow-up, as well as in the left eye at 1 month (G) and 6 months (H). Perfusion deficit of the right eye is noted at the area of the lesion in the choriocapillaris (C) and choroid (E), with choroidal vascular dilatation (arrowhead). Similar areas of choriocapillaris (I) and choroidal (K) hypoperfusion is seen in the fellow eye. Improved perfusion is noted at 6-month follow-up in both eyes (D, F, J, L), with persistent choroidal vascular dilatation in the right eye (F, arrowhead).

SD-OCTA of the right eye was obtained one month after the initial presentation and demonstrated normal-appearing retinal circulation, including deep capillary plexus (DCP). Notably, there was a vascular perfusion defect at the level of the choriocapillaris and choroid in the area corresponding to the lesion (Figure 2). Similar involvement of the choriocapillaris with sparing of the DCP was also seen on SD-OCTA slabs of the left eye, corresponding to areas of hypocianescence seen on late-phase ICG.

The patient was found to have strongly positive IgG titers for coxsackievirus A7 (1:800), A9 (1:1600), A64 (1:1600), and A24 (1:1600). Respective IgM titers were negative. An extensive laboratory workup was otherwise normal. An oral prednisone (1 mg/kg) taper was initiated, and the patient was monitored closely. Six months after initial presentation, patient's subjective complaints of a scotoma had resolved and SD-OCTA revealed improved perfusion of the choriocapillaris and choroid and OCT showed improvement in the attenuation of the ellipsoid zone (Figures 1(b) and 2).

## 3. Discussion

Acute idiopathic maculopathy is a rare, self-limited, macular disease disproportionately affecting young men [1]. It has been postulated that AIM exists on a spectrum of acute multifocal placoid pigment epitheliopathy (APMPPE) [5] and our case illustrates this point. The lesions in APMPPE clinically appear yellow-white and may have a complex outer boundary due to confluence of smaller lesions. In contrast, the lesion in our case is solitary with an ovoid shape and smooth border. Despite the lack of classically described subretinal fluid or neurosensory detachment, the angiographic and autofluorescence findings were consistent with previously reported cases of AIM [2, 3]. In this report, early arteriovenous phase FA showed hyperfluorescence of the lesion and a staining pattern in the late recirculation phase. This is in contrast to APMPPE in the acute stage, which reveals an intense, homogeneous hypofluorescence at the site of the placoid lesions. The FAF in this case was similar to findings in APMPPE and demonstrated a mixed pattern of hyperautofluorescence corresponding to deposition of lipofuscin from RPE cell thickening or duplication and hypoautofluorescence from RPE cell loss [6].

This case further supports the growing speculation that the pathogenesis of AIM is primarily a choroiditis with the changes to overlying RPE and outer retina occurring secondarily to choroidal ischemia [7–9]. The multiple, bilateral areas of choroidal hypoperfusion, as demonstrated by moth-eaten areas of hypocianescence on ICG, also point to a primary choroidal insult. More impressively, SD-OCTA shows hypoperfusion in the choroid and choriocapillaris of both eyes, without involvement of the DCP in the subacute phase—similar to findings in APMPPE [10, 11]. Lee et al. also concluded a primarily choroidal insult in acute macular neuroretinopathy by studying OCTA findings but noted that choriocapillaris flow loss persisted [12]. After 6 months of follow-up, we observed a partial return of choroidal perfusion voids, with concomitant resolution of visual symptoms. Hashimoto et al. demonstrated a similar change in choroidal perfusion with recovery in cases of AIM [8] as well as APMPPE [11], further suggesting an overlap between these diseases.

The relatively good visual recovery and reconstruction of the outer retinal layers in this case suggest that the disease process is reversible in AIM. Jung and colleagues hold that the preservation of the external limiting membrane may indicate that the inner retina, cell nuclei, and cellular structures necessary for outer segment regeneration remain intact [13]. Furthermore, restoration of the RPE pump function likely occurs following resolution of the acute inner choroidal inflammatory process.

Multiple reports have documented positive coxsackievirus serologies in association with AIM [2, 3]. In our case, the patient endorsed a viral prodrome prior to visual symptoms and was found to have strongly positive IgG titers for coxsackievirus A7, A9, A64, and A24. Whether due to direct viremia or by an aberrant response due to molecular mimicry in the setting of viral infection, the exact mechanism by which coxsackievirus causes tissue insult remains unclear.

Limitations of any case report include overinterpretation and an inability to imply a cause-effect relationship. In this report, the interval from viral illness and onset of eye symptoms was 12 weeks—a longer lag time than is usually seen. The positive coxsackie IgG titers are only suggestive of an association but not definitive. Lastly, the OCTA images were obtained one month after the onset of symptoms, allowing time for vascular reorganization.

## 4. Conclusion

We present a case of AIM and use multimodal imaging including OCTA as well as serologic testing to make the challenging diagnosis. Our findings suggest that the pathogenesis of AIM involves a primary insult to the inner choroid with secondary RPE and outer retinal damage, similar to the mechanism suggested in APMPPE [7–9, 11].

## Data Availability

The case report data used to support the findings of this study are included within the article.
